# Characterization of urethra closure in female neonatal mice at histological and molecular levels

**DOI:** 10.1530/REP-24-0239

**Published:** 2024-09-26

**Authors:** Abigail S Kitakule, Ciro M Amato, Humphrey Hung-Chang Yao

**Affiliations:** 1 Reproductive Developmental Biology Laboratory, National Institute of Environmental Health Sciences, Research Triangle Park, Durham, North Carolina, USA; 2 Department of Surgery, Division of Urology, University of Missouri, Columbia, Missouri, USA

## Abstract

**In brief:**

Female hypospadias is a little-known and poorly studied birth defect. This research establishes an anatomical and molecular foundation for future research to investigate the origins of this defect.

**Abstract:**

Hypospadias is a congenital anomaly of the external genitalia where the urethra does not properly close. In humans, hypospadias is mostly reported in male newborns, whereas in females hypospadias is rare, although it is generally considered to be under-reported. Improper urethra closure in the female genitalia can cause recurrent genitourinary tract infections and infertility. In mice, female hypospadias was induced by exposure to exogenous estrogenic compounds. Aside from the link between estrogen exposure and female hypospadias, the process of female urethra closure is largely unstudied, with the precise timing of urethra closure and associated molecular mechanisms remaining poorly understood. To address this gap, we determined when urethra closure occurs and identified gene expression patterns during the process of urethra closure in female neonatal mice from postnatal day (PND) 5 to 10. Using whole mount imaging and histology, we discovered that the initiation of urethra closure begins at PND7, and urethra closure is fully completed by PND10. To identify the genes associated with urethra closure, we conducted bulk RNA sequencing on female external genitalia prior to and after urethra closure. Gene ontology analyses revealed an increase in steroidogenic gene expression (*Star, Hsd3b6,* and* Cyp17a1)* during urethra closure, suggesting that the female genitalia locally produce steroids which could facilitate steroid signaling within the genitalia. With this study, we establish an anatomical timeline of female urethra closure and hypothesize a paracrine steroid signaling mechanism of urethra closure. These observations provide entry points to aid in further understanding external genital abnormalities, like hypospadias, in females.

## Introduction

Birth defects have profound effects on millions of newborns worldwide (Swanson *et al.* 2023). These anomalies not only pose challenges to the health and well-being of the affected individual but can also carry far-reaching financial and emotional implications for their close relatives (Cole *et al.* 2015, 2018, Soulvie *et al.* 2012). Some of the most common birth defects in the world are of the external genitalia. Hypospadias, which is defined as improper urethra closure in the genitalia, is a particularly common birth defect that affects approximately 1:125 male newborns (Paulozzi *et al.* 1997, van der Horst & de Wall 2017). Female hypospadias is considered rare; however, it is thought that female hypospadias is drastically under-reported due to a lack of investigation by general practitioners (Knight *et al.* 1995, Tug *et al.* 2020). As a result, the basic genetic and hormonal mechanisms of female urethra closure are largely unstudied.

Female hypospadias is a structural abnormality of the urethra, where the urethral meatus is located on the anterior vaginal wall as opposed to above the vaginal opening (Hill *et al.* 1982). This defect is a result of failed urethra fold fusion and is usually coupled with other female genitourinary abnormalities like vaginal septum, vaginal atresia, and urogenital septum (Prakash *et al.* 2016). Studies using mice have shown a similar suite of abnormalities in cases of female hypospadias (Miyagawa *et al.* 2001, Padilla-Banks *et al.* 2011). While the molecular processes underlying the disruption of normal urethra closure remain unclear, it is noteworthy that between birth and puberty, circulating estrogens, progesterones, and androgens are low or undetectable in the serum of female mousee pups (Bell 2018). These data suggest that systemic steroids may not be involved in female urethra closure. However, female mice exposed to exogenous endocrine-disrupting chemicals such as diethylstilbestrol (DES), genistein, and tamoxifen consistently developed hypospadias, cleft clitoris, and premature vaginal opening (Taguchi & Nishizuka 1985, Miyagawa *et al.* 2001, Padilla-Banks *et al.* 2011). On the other hand, female mice exposed to progesterone during this same time displayed no changes in urethra closure and overall clitoral morphology (Iguchi & Takasugi 1976). Also, female mice exposed to androgens from birth to postnatal day 5 displayed drastic increases in the size of the clitoral body and accelerated urethra closure (Cunha *et al.* 2023). It is evident that the perinatal mouse external genitalia are sensitive to steroids, even in the lack of circulating steroid hormones. It remains to be determined whether steroids are involved in normal urethra closure.

In this study, we aimed to pinpoint the developmental timing and molecular pathways governing urethra closure in female neonatal mice. Our first goal was to precisely determine the developmental timeline of urethra closure initiation and completion within the female external genitalia by using histological analysis and whole mount imaging. We then identified the gene expression differences during urethra closure using bulk RNA sequencing prior to and after urethra closure. This study takes important steps to identify the critical mechanisms involved in female urethra closure.

## Materials and methods

### Animals

C57BL/6J mice, aged 2–5 months were time-mated with male C57BL/6J mice, and the observation of a vaginal plug was considered embryonic day or E0.5. Female mice were then separated from males and were allowed to give birth to pups. Pups were collected at postnatal days 6, 8, and 10. Pups were euthanized with CO_2_ asphyxiation and decapitation as secondary euthanasia. The pups were sexed by evaluating the anogenital distance and genital morphology. Pups were then weighed using an Ohaus Explorer analytical balance (EX124) (Ohaus Corporation, Parsippany, NJ, USA), and the anogenital distance was measured with calipers. Genitalia were collected for RNA extraction, bulk-RNA sequencing, immunofluorescence, and histology. All animal procedures were approved by the National Institute of Environmental Health Sciences (NIEHS, Triangle Park, NC, USA) Animal Care and Use Committee and followed a NIEHS-approved animal study proposal.

### Immunofluorescence and histological analysis

Female external genitalia were collected via dissection following euthanasia, fixed in 4% paraformaldehyde overnight at 4°C, and washed in 1X PBS three times for 10 min the following day. The samples were dehydrated through an ethanol gradient and then embedded in paraffin wax. The samples were sectioned at 8 μm thickness and mounted onto charged microscope slides (Fisher Scientific, 12-550-15, Pittsburgh, PA, USA). For immunofluorescence, sections were dewaxed and rehydrated in an ethanol gradient. The sections on slides were pretreated in 0.1 mM citrate-based Antigen Unmasking Solution for 20 min in a microwave at 10% power and then cooled to room temperature. The sections were blocked with 5% normal donkey serum in 1X PBS Triton X-100 solution for 1h and incubated with primary antibodies overnight at 4°C (Supplementary Table 1, see section on supplementary materials given at the end of this article). The following primary antibodies were applied: anti-Progesterone Receptor A/B (1:1000 #8757, Cell Signaling Technology, Danvers, MA, USA), anti-Estrogen Receptor alpha (1:100; sc-7207, Santa Cruz Biotechnology, Santa Cruz, CA, USA), anti-Androgen Receptor (1:200; ab133273, Abcam, Cambridge, MA, USA), anti-StAR (1:200; ab203193, Abcam), and anti-CYP17A1 (1:400; sc-46081, Santa Cruz). The sections were then washed with 1X PBS-Triton X-100 and incubated with 488, 568, and 647 Alexa-Fluor secondary antibodies for 1h at room temperature. The sections were washed again with 1X PBS-Triton twice and 1:1000 dilution of DAPI in PBS. The slides were then mounted in ProLong Diamond Antifade Mountant (Invitrogen, Waltham, MA, USA) and imaged under a Zeiss LSM900 (Oberkochen, Germany) confocal microscope using the Zen software (Oberkochen, Germany).

For histological analysis, the slides were deparaffinized and rehydrated through an ethanol gradient before being stained in Harris hematoxylin and counterstained in eosin. Slides were then dehydrated, cleared, and mounted with Permount. The sections were imaged under the Keyence BZ-X810 fluorescence microscope (Keyence, Osaka, Japan) using a 20× objective lens magnification.

### RNA extraction

To investigate the molecular processes of female closure, bulk RNAseq was used. Postnatal day 6, 8, and 10 female external genitalia were collected via dissection, snap-frozen, and stored at −80°C. RNA extractions were conducted using three biological replicates at each developmental time point. To homogenize the samples, a Tissue Tearor homogenizer was used in 30 s increments until the tissue was fully dissolved in the extraction buffer. The homogenate was then processed with the Arcturus PicoPure RNA Isolation Kit (Thermo Fisher Scientific, Waltham, MA, USA) per the manufacturer’s instructions. The stock concentration for each sample was determined using the Qubit 2.0 Fluorometer (Invitrogen), and RNA quality was determined using the Agilent RNA ScreenTape System (Agilent Technologies, Santa Clara, CA, USA). All samples had an RIN value greater than 8.8.

### Bulk RNA sequencing and analysis

Bulk mRNA Sequencing was performed on P6, P8, and P10 female external genitalia using the Illumina TruSeq Stranded mRNA library preparation kit. 250 ng of isolated RNA was used for each replicate for library preparation. Libraries were sequenced on the Illumina NextSeq550 with paired-end sequencing and a 150 bp read length. Libraries were sequenced to a depth between 29 to 57 million reads (Supplementary Table 1; sequencing data can be found on GEO accession number GSE275833). Fastq files were assessed for quality with FastQC and then were aligned and analyzed for differential gene expression. Library preparation and bulk RNA sequencing were performed by the Epigenomics and DNA Sequencing Core Facility at NIEHS.

### Statistical analysis

After sequencing, fastq files were pseudo-aligned with kallisto, using the mm38 mouse genome as a reference (Bray *et al.* 2016). Resulting files were then transferred into the R statistical environment, where DEseq2 was used to conduct differential expression analysis (Love *et al.* 2014). Standard filters for genes with low transcript counts were equally applied to each dataset. Pairwise comparisons were made for P8 vs P6, P10 vs P6, and P10 vs P6. Genes with an adjusted *P*-value < 0.05 were considered differentially expressed genes. Ingenuity pathway analysis (IPA) was then conducted on the differentially expressed gene lists to obtain gene ontologies and upstream regulators. All heatmap visualizations were conducted with pheatmap (Kolde 2019).

## Results

### Determining the timing of initiation and completion of urethra closure in female neonatal mice

**Figure 1:**
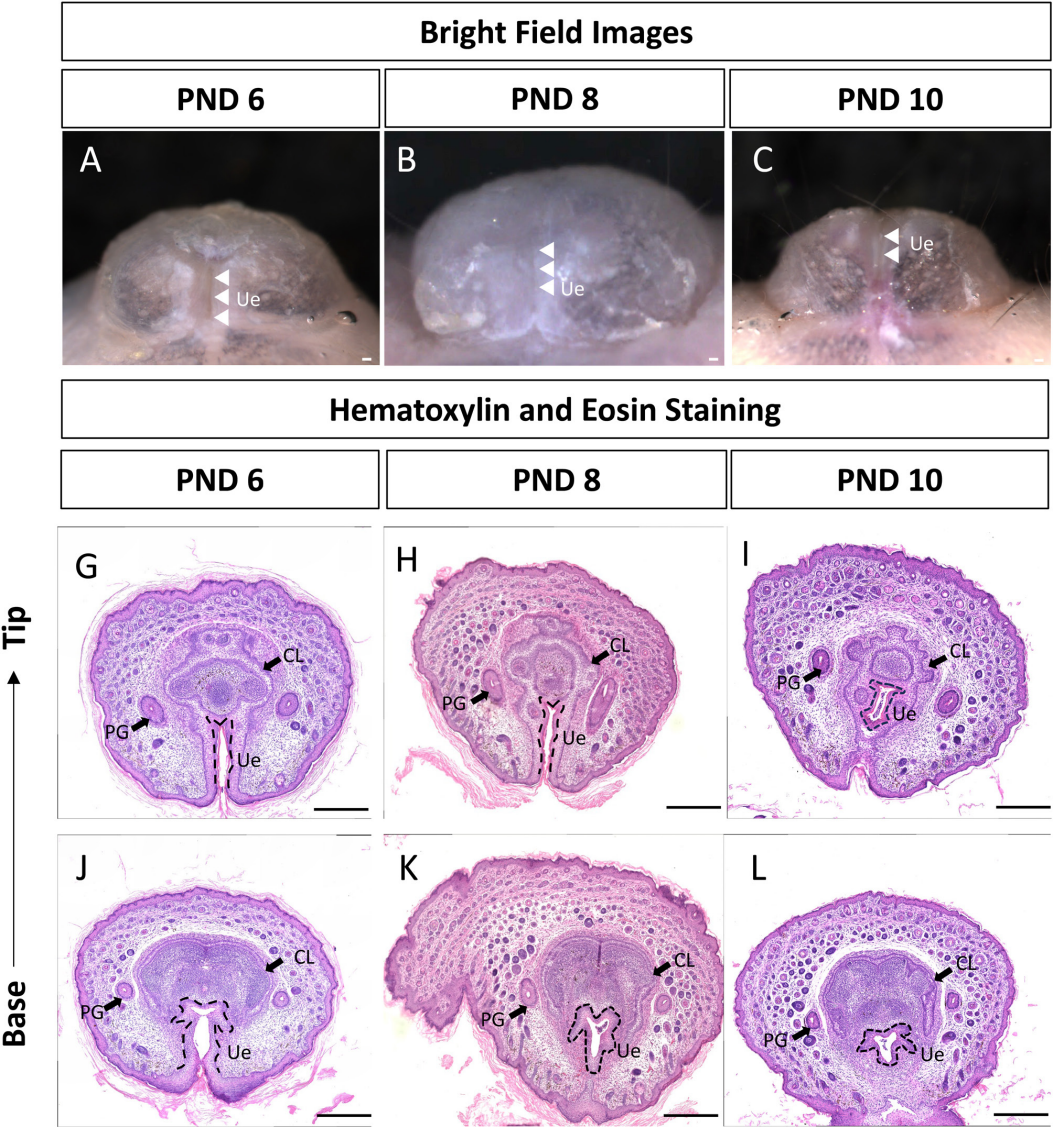
The timing of initiation and completion of urethra closure in the female genitalia. (A–C) Whole mount images of female genitalia at PND6 (A), PND8 (B), and PND10 (C). White arrows indicate the positioning of the open urethra. (G–L) Hematoxylin and Eosin staining of the developing female genitalia. (G–L) are distally located sections, while J–K are proximally located sections. The dotted line outlines the urethral epithelium. CL, clitoral lamella; PG, preputial gland: UE, urethral epithelium. Scale bars in panels A–C are 100 µm and panels D-I are 250 µm.

To understand the process of urethra closure, it is important to identify critical developmental landmarks. It was documented that urethra closure in female mice is completed by PND10; however, when urethra closure starts is not known (Cunha *et al.* 2020). To precisely determine the time of female urethra closure, we examined female mice from PND6-10. Based on the whole mount images, the urethra remained completely open from the base to the tip of the female external genitalia at PND6 (Fig. 1A and D). This observation was corroborated by hematoxylin and eosin staining of histological sections (Fig. 1G and J). At PND8, the urethra started to close at the base of the female genitalia but remained open in the middle of the genitalia (Fig. 1B and E). In histological sections, the urethra was closed in the base sections where it began to be pushed back toward the u-shaped clitoral lamina but remained open in the tip section of the genitalia (Fig. 1H and K). By PND10, the exit of urethra was observed at the tip of the genitalia (Fig. 1C, F, I, and L). Based on these data, we conclude that the urethra closure in female mice starts around PND8 in a base-to-tip wave, and the urethra becomes fully closed by PND10.

### Defining general gene expression trends in female external genitalia

**Figure 2:**
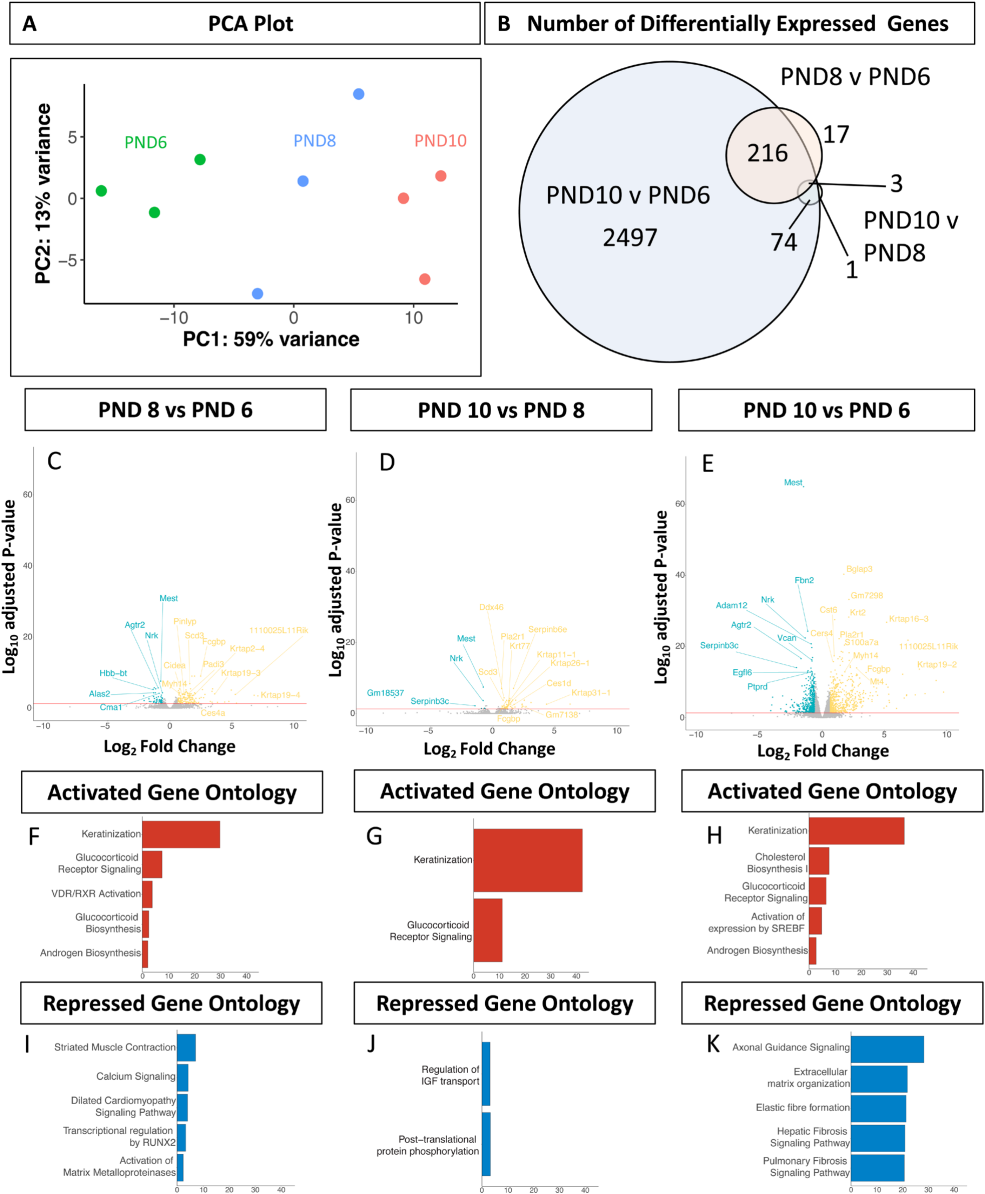
Identifying gene expression trends in the female genitalia. (A) Principal component analysis or PCA of the developing female genitalia at PND6 (green), PND8 (blue), and PND10 (red). (B) Venn diagram of differentially expressed genes identified in PND6 vs PND10, PND8 vs PND6, and PND10 vs PND8 comparisons. (C–D) Volcano plots of differentially expressed genes found in each pairwise comparison, with green color representing downregulated genes and yellow color representing upregulated genes. (F–G) Gene ontologies for all genes upregulated at later time points for each pairwise comparison. The x-axis represents -log(fold-change). (I–K) Gene ontologies for all genes that are downregulated at later time points.

With the precise timing of urethra closure established, we used bulk mRNA sequencing to characterize the gene expression changes in the female external genitalia during the process of urethra closure. External genitalia were collected prior to urethra closure (PND6), during urethra closure (PND8), and after urethra closure (PND10) and processed for differential gene expression analysis. Each developmental time point had distinct gene expression profiles from the other time points and had a clear, time-dependent developmental trajectory (Fig. 2A). The transcriptomes of PND6 external genitalia were more different from those of the PND10 than the PND8 genitalia (Fig. 2A). To determine how different each stage was from each other, we conducted a differential gene expression analysis and found that PND10 vs PND6 transcriptomes had the greatest number of differentially expressed genes or DEGs (2787 genes; Fig. 2B and E), followed by 236 DEGs between PND8 vs PND6 and 78 DEGs between PND10 vs PND8 (Fig. 2B–D). The overlap in DEGs between PND10 vs PND6 and the other two pairwise comparisons was high. 93% (216 DEGs) of DEGs found in the PND8 vs PND6 comparison were found in the PND10 vs PND6 comparison, and 99% (77 DEGs) of DEGs from the PND10 vs PND8 comparison were found in the PND10 vs PND6 comparison (Fig. 2B). Only 1% (3 DEGs) of DEGs from the PND10 vs PND8 were found in the PND8 vs PND6 comparison. These same DEGs were also identified in the PND10 vs PND6 comparison. This comparison of DEG overlap shows that there are consistent transcriptional changes that occur from PND6 through PND10, with the changes from PND6 to PND8 being more dramatic than changes from PND8 through PND10

To identify the general signaling pathways occurring throughout female urethra closure, we conducted Gene Ontology analysis on genes that either increased or decreased expression through developmental time. Significant ontologies from genes that increased through development were classified as ‘activated gene ontologies’ and significant ontologies from down-regulated genes were denoted as ‘repressed gene ontologies’. In the activated gene ontologies for P10 vs P8, P8 vs P6, and P10 vs P6 comparisons, ‘Keratinization’ was the most active gene expression network (Fig. 2F-H). This is driven by the abundance of *Krtap* and *Krt* mRNA, which are involved in keratinization of the skin and hair follicle development (Fig. 2C-E) (Khan *et al.* 2014, Ho *et al.* 2022). The next most common signaling pathways were related to nuclear receptor signaling (Fig. 2F-H). Glucocorticoid Receptor Signaling was identified in P10 vs P8, P8 vs P6, and P10 vs P6 and had many common steroid-responsive genes, like *Klk3, Zeb1,* and* Stc1* (Fig. 2F-H). Following nuclear receptor signaling was steroid biosynthesis, with androgen iosynthesis being found in both the PND8 vs PND6 and PND10 vs PND6 comparisons (Fig. 2F and H). The major DEGs that contributed to these ontologies were steroidogenic enzymes *Hsd3b6, Cyp17a1, Srd5a1, Srd5a2,* and others.

Next, we investigated the gene ontologies that are associated with decreased gene expression through development. In the repressed gene ontology analysis, there were many pathways associated with cell migration and extracellular matrix modifications (Fig. 2I-K). In the PND8 vs PND6 comparison pathways related to muscle contraction, calcium signaling, and metalloproteases were downregulated, while in the PND10 vs PND8 comparison, pathways related to IGF transport and protein phosphorylation were downregulated. The PND10 vs PND6 comparison showed abundant downregulation of cell migration and extracellular matrix-related pathways. Overall, these data indicate that gene expression is actively changing during urethra closure with an upregulation of keratinization, steroid signaling, and steroid synthesis, and a downregulation of cell migration and ECM modifications during female urethra closure.

### Steroid receptors are expressed and active during female urethra closure

The findings of steroid signaling and biosynthesis during female urethra closure, coupled with previous findings on the negative impacts of endocrine-disrupting chemicals on urethra closure, led us to hypothesize that steroids could be involved in female urethra closure. Steroid receptors are transcription factors that regulate the expression of a vast array of genes. To predict which steroid receptors are involved in female urethra closure, we conducted an IPA upstream analysis of the DEGs from the P10 vs P6 comparison. This analysis revealed that steroid receptors, including estrogen receptors (ER) and progesterone receptors (PR), as well as the ligand for androgen receptors (AR), 5α-dihydrotestosterone, were predicted to be upstream of the DEGs identified in the sequencing data. In addition to providing predicted transcriptional regulators, the upstream analysis also provides information on whether the transcriptional regulator is being activated nor inhibited. An activated transcriptional regulator has gene expression changes that are concordant with existing literature. An inhibited transcriptional regulator has gene expression changes that are opposite to the changes presented in the literature. The upstream analysis showed that the estrogen signaling pathway was mostly inhibited in expression, whereas progesterone signaling pathway was activated, and the 5α-dihydrotestosterone (5α-DHT) pathway was neither activated nor inhibited. Neither *Esr1*, *Pgr*, nor *Ar* were differently expressed at the RNA levels among the different developmental stages.

**Figure 3:**
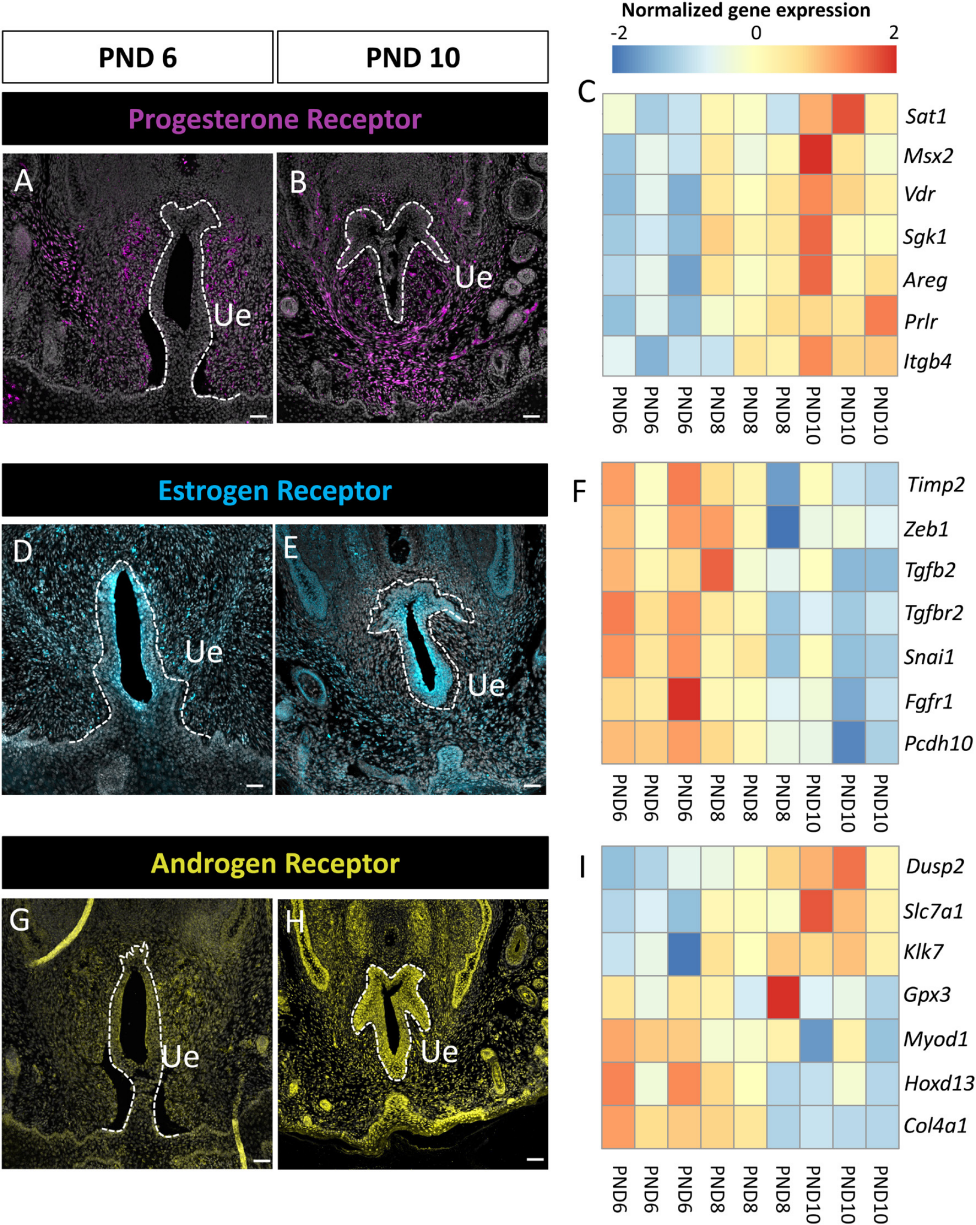
Expression of steroid receptors and their associated genes during female urethra closure. (Aand B) Immunofluorescence for PR at PND6 and PND10 in the female genitalia with the white dotted line indicating the urethra epithelium (UE). (C) Heatmap of progesterone-related gene expression for each individual sample, with blue indicating lower expression and red indicating elevated expression. (Dand E) Immunofluorescence for ERα at PND6 and PND10 in the female genitalia. (F) Heatmap of estrogen responsive genes. (G and H) Immunofluorescence for AR at PND6 and PND10 in the female genitalia. (**I**) Heatmap of androgen-responsive genes. Scale bars in panels A, B, D, E, G, and H are 100 µm.

To determine where these steroid receptors are present in the female external genitalia during urethra closure, we performed immunofluorescence on female genitalia prior to urethra closure at PND6 and after urethra closure at PND10. PR was detected in female external genitalia at both PND6 and PND10 (Fig. 3A and B). At PND6, PR-expressing cells were localized in the mesenchyme on opposite sides of the open female urethra epithelium (Fig. 3A). However, the cells immediately adjacent to the urethral epithelium had only cytoplasmic, not nuclear, localization, in contrast to the cells lateral to this population of cells. At PND10, PR-expressing cells occupied the space underneath the closed urethral tube (Fig. 3B). Consistent with the presence of PR proteins, we found significant upregulation of progesterone-responsive genes *Sat1, Msx2, Vdr, Sgk1, Areg, Prlr, Itgb4,* and others, implicating that progesterone signaling is active during urethra closure (Fig. 3C).

ERα or ESR1 protein expression was sparsely found throughout the female external genitalia at both PND6 and PND10 and had nuclear localization in the urethral epithelium and a small subset of the surrounding mesenchymal cell populations (Fig. 3D and E). When investigating the gene expression data, critical estrogen-responsive genes, *Timp2, Zeb1, Tgfb2, Tgfbr2, Snai1, Fgfr1, Pcdh10,* and others were downregulated in expression (Fig. 3F). Based on literature, these genes are commonly activated by ERα signaling (Roy & Kole 1995, Kowase *et al.* 2007, Ariazi *et al.* 2011, Notas *et al.* 2013).

Androgen signaling is required for normal male urethra closure. Exogenous androgen can induce urethra closure in female embryos. At PND 6, we found low-level nuclear staining of AR throughout the female genitalia (Fig. 3G and H). At PND10, nuclear AR protein was detected throughout most cell populations in the female genitalia (Fig. 3I). There was mixed regulation of androgen-responsive genes, with *Dusp2, Slc7a8,* and *Klk7* being upregulated and *Gpx3, Myod1, Hoxd13,* and *Col4a1* being downregulated. Altogether, these data demonstrated that AR, ERα, and PR are all expressed within the female external genitalia. Each receptor has different localization within the female genitalia.

### The female external genitalia expresses critical steroidogenic enzymes during urethra closure

**Figure 4:**
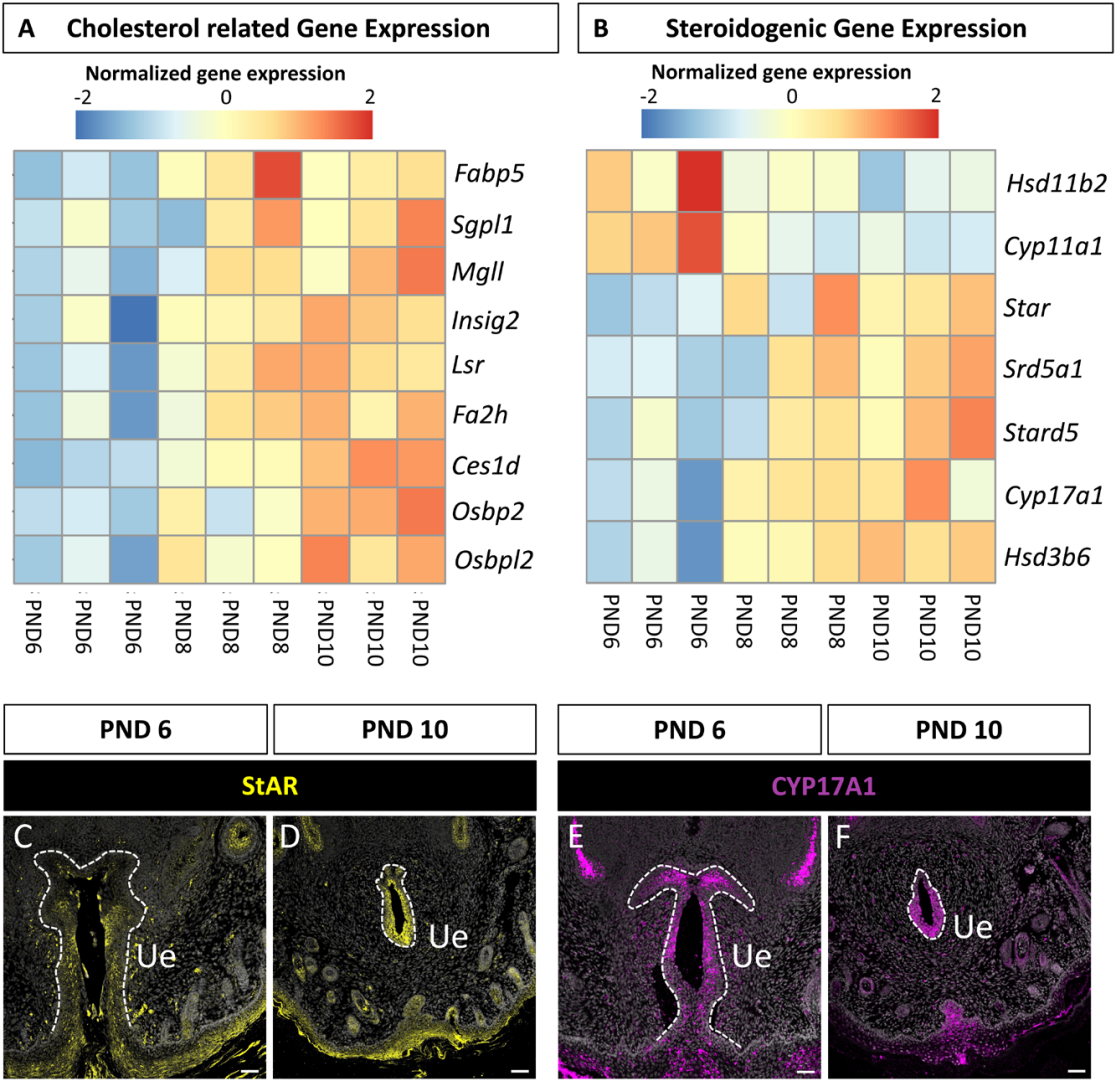
Steroidogenic gene expression in the female external genitalia (**A**and **B**) Heatmaps of cholesterol biosynthesis and steroid biosynthesis gene expression for each sample during female urethral closure. Blue indicates lower levels of expression and red indicates high levels of expression. (**C-F**) Immunofluorescence for StAR (C and D) and CYP17A1 (E and F) at PND6 and PND10 in the female genitalia. White dotted lines indicate the urethral epithelium. UE = urethral epithelium.

Progesterone, estrogen, and androgen levels in the perinatal circulation are quite low after birth in mice (Bell 2018). Although gonads are the major source of circulating steroids, other tissues, like adipose and immune cells, have the capacity to produce steroids locally for site-specific signaling (Ito *et al.* 2005, Li *et al.* 2015*a*, Chakraborty *et al.* 2021). We examined whether the female external genitalia have the capacity to undergo steroidogenesis. Steroidogenesis requires cholesterol as a precursor molecule for downstream enzymatic processes. We found there is an extensive amount of cholesterol biosynthesis genes that are upregulated during the urethra closure time window (Fig. 4A). One of the pathways downstream of cholesterol synthesis is steroidogenesis. The initial step to steroid production is to import cholesterol into mitochondria through the StAR protein. In the bulk mRNA dataset, *Star* mRNA expression increased during the process of urethra closure (Fig. 4B). StAR proteins were found to be expressed within the urethra epithelium and the skin (Fig. 4C and D). The next critical step of steroidogenesis is the cleavage of cholesterol into pregnenolone by CYP11A1.* Cyp11a1* mRNA was significantly downregulated in the female genitalia during urethra closure (Fig. 4B). After pregnenolone conversion, a variety of enzymes are responsible for the synthesis of a diverse array of steroids (Connan-Perrot *et al.* 2021). *Hsd3b6,* the critical enzyme that converts pregnenolone to progesterone, was significantly upregulated at the mRNA level in the female genitalia (Fig. 4B). We also observed an increase in mRNA expression of the critical enzymes for androgen synthesis, *Cyp17a1* and *Srd5a1,* during the process of urethra closure (Fig. 4B). CYP17A1 protein expression was mostly localized to the urethral epithelium and skin (Fig. 4E and F). Estrogens are generated from androgen precursors through the enzyme CYP19A1. *Cyp19a1* mRNA was not identified as a DEG, and it was found that *Cyp19a1* was exceedingly low in all genitalia samples. These data suggest that the female external genitalia have the capacity to produce progesterones and androgens locally, but not estrogens.

## Discussion

In this study, we characterized the urethra closure in female neonatal mice. The female urethra remains completely open until PND6. By PND8, the urethra initiates closure in a proximal-to-distal wave, with the urethra being closed at the base of the genitalia and remaining open at the tip of the genitalia. At PND10, the urethra is fully closed along the external genitalia. Using bulk mRNA sequencing, we identified general patterns of gene expression changes during female urethra closure. We found that steroid signaling is abundant during female urethra closure. Both PR and AR are abundantly present in the genitalia during urethra closure. In the urethral epithelium, there are critical steroidogenic genes that become highly expressed as the urethra closes. Steroids produced within the urethra are likely the major contributors to steroid signaling.

### Differences and similarities of female and male urethra closure

Urethra closure in mice has been studied more extensively in males than females. Several similarities and differences can be noted between the two sexes. The most apparent difference is that the urethra closure in male mice initiates during embryonic development (E14.5–18.5) while the female urethra closes several days later during postnatal development (Georgas *et al.,* 2015). Despite the drastic time difference in closure, the pattern of closure from proximal to distal along the shaft of the external genitalia is similar between males and females (Li *et al.* 2015*b*, Wang *et al.* 2018). In males, this process is tightly stimulated by proper androgen signaling: loss of androgen signaling causes severe cases of hypospadias (Zheng *et al.* 2015). In the male mice, testosterone is largely derived from the embryonic testis, and testosterone is converted to the more potent androgen 5α-DHT by the enzyme 5α-reductase in the external genitalia (O'Shaughnessy *et al.* 2019). During human penis development, 5α-DHT is thought to be synthesized from 7OH-progesterone through the ‘back-door DHT synthesis pathway’ (O'Shaughnessy *et al.* 2019). The penis expresses the enzymes *Akr1c2* and 5α-reductase, which allow for this site-specific conversion of 5α-DHT from a non-androgenic chemical (O'Shaughnessy *et al.*2019). In the female genitalia, exposure to 5α-DHT at PND0-PND5 accelerated the rate of urethra closure so it was fully closed by PND5 (Cunha *et al.* 2023). This is five days before the female urethra is closes. Female pups have not been exposed to anti-androgens to determine whether androgens are essential for normal female urethra closure. We found the female genitalia express critical genes in steroidogenesis such as *Star, Hsd3b6, and Cyp17a1*. Both *Cyp17a1* and *Star* were specifically expressed within the urethral epithelium, suggesting that the urethral epithelium may serve as a steroid source in the female external genitalia.

In the male mouse, cell migration is critical for the process of urethra closure. The main cell populations in male urethra closure interact through cell migration pathways and extracellular matrix modifications (Acebedo *et al.* 2019, Amato & Yao 2021, Alcantara *et al.* 2022). An overall increase in the expression of cellular migration-related genes and extracellular matrix genes was observed during male urethra closure (Amato & Yao 2021). In female mice, in contrast to the male, there is an overall decrease in the expression of cell migration-related genes and extracellular matrix genes. This suggests that developmental processes that lead to urethral closure differ between males and females.

### Steroidal control of female urethra closure

We found that ERα, PR, and AR signaling were some of the most prevalent pathways found in mRNA sequencing data during female urethral closure. Genes associated with the estrogen signaling pathway were inhibited, while genes associated with the progesterone signaling pathway were activated and the androgen signaling pathway was intermediate. Steroid receptors are well known to either synergistically or antagonistically interact with each other (Mohammed *et al.* 2015, Wu *et al.* 2017, Michmerhuizen *et al.* 2020). In the mouse uterus, there is an antagonistic relationship between progesterone and estrogenic signaling (Tibbetts *et al.* 1999). Progesterone and estrogen signaling in the uterus are regulated by their respective ligands. ERα signaling positively regulates its own expression and that of PR, while progesterone signaling represses its own expression and the expression of ERα (Wang *et al.* 2017). A similar type of antagonism is found between AR and ERα. Androgen exposure can inhibit the effects of estrogen, while estrogen exposure can inhibit androgen signaling (Michmerhuizen *et al.* 2020). Indeed, estrogenic compounds such as DES and genistein cause hypospadias in both male and female mice during development (Suk Kim *et al.* 2004, Vilela *et al.* 2007, Stewart *et al.* 2018). Under normal conditions, estrogen, androgen, and progesterone may act in balance, but in hypospadias conditions, the scale may be tipped towards excessive estrogen signaling, which suppresses the proper progesterone or androgen signaling.

Birth defects affecting the female external genitalia, such as hypospadias, are rare and often unreported. The precise cause of female hypospadias remains elusive due to gaps in our understanding of normal female urethra development. Our findings in this study lay the foundation for studying female urethra closure by defining an anatomical timeline for urethra development and identifying pathways potentially involved in the process. These insights will advance our comprehension of external genital abnormalities such as hypospadias. Moreover, by raising awareness of this rare anomaly, our findings contribute to the broader efforts to address and mitigate the impact of congenital malformations affecting the female reproductive system.

## Supplementary Materials

Supplemental Table 1

## Declaration of interest

The authors declare that there is no conflict of interest that could be perceived as prejudicing the impartiality of the research reported.

## Funding

This work was supported by the Intramural Research Program of the NIH, National Institute of Environmental Health Scienceshttp://dx.doi.org/10.13039/100000066 (ES102965 to HH-CY), NIEHS Scientific Director’s Traineeship for Inclusion, Diversity, and Equality (STrIDE to AK), and National Institute of Diabetes and Digestive and Kidney Diseaseshttp://dx.doi.org/10.13039/100000062 (DK132460-01 to C.M.A).

## Author contribution statement

ASK - Conceived the study, conducted the experiments, and wrote the article. CMA - Conceived the study, conducted the experiments, analyzed the data, provided constructive feedback, and edited the article. HH-CY - Edited the paper and provided constructive feedback.
