# Supplementary material for: Characterization of urethra closure in female neonatal mice at histological and molecular levels
Source: Reproduction. 2024 Sep 26;168(5):e240239. doi: 10.1530/REP-24-0239 (PMC11427134; doi:10.1530/REP-24-0239)
Supplement: Supplemental Table 1 [file supplementary_table_1.pdf]

Supplemental Table1

Sample	Sequencing Depth
P6	31,477,011
P6	44,130,362
P6	36,898,288
P8	29,917,359
P8	31,193,383
P8	54,740,825
P10	41,463,580
P10	37,821,997
P10	47,181,416
